# Prognostic value of blood urea nitrogen to albumin ratio in septic patients with acute kidney injury—a retrospective study based on MIMIC database

**DOI:** 10.3389/fmed.2025.1510919

**Published:** 2025-05-07

**Authors:** Kun Han, Yuxia Tao, Jianhao Wang, Jinshuai Lu

**Affiliations:** ^1^Postgraduate School, Xinjiang Medical University, Urumqi, China; ^2^Xinjiang Uygur Autonomous Region People’s Hospital Emergency Center, Urumqi, China

**Keywords:** sepsis, sepsis-induced acute kidney injury, blood urea nitrogen to albumin ratio, mortality rate, nomogram

## Abstract

**Objective:**

To investigate the predictive value of blood urea nitrogen to albumin ratio (BAR) in the prognosis of patients with sepsis-induced acute kidney injury (S-AKI).

**Methods:**

A retrospective analysis was conducted on patient data from the MIMIC-IV database that met the S-AKI criteria. Cox regression was employed to analyze the relationship between BAR and 28-day mortality risk. BAR was divided into four quartiles (Q1, Q2, Q3, Q4), and Kaplan-Meier survival analysis was performed to compare the 28-day cumulative survival rates among the four patient groups. Simultaneously, the log-rank test was used for statistical analysis of survival rate differences among the four groups. Subsequently, Cox regression was performed with Q1 (the lowest quartile) as the reference for comparison. Restricted cubic splines (RCS) were utilized to analyze the non-linear association between BAR and mortality risk, with the median BAR of all patients serving as the reference point to define the non-linear effect. Thereafter, correlation analysis and subgroup analysis were conducted to assess the stability of BAR in predicting 28-day prognosis. LASSO regression analysis was applied to select variables related to 28-day prognosis, and relevant variables were screened through univariate and multivariate logistic regression analyses to construct a nomogram model. The area under the receiver operating characteristic curve (AUC), calibration plot, and decision curve analysis (DCA) were used to evaluate the predictive performance of the nomogram for in-hospital mortality in S-AKI patients.

**Results:**

A total of 8,666 patients with S-AKI were included, among whom 2,396 died (27.65%). Cox analysis of BAR indicated a positive correlation between BAR and 28-day mortality risk, with an HR of 1.029 (95% CI: 1.026-1.032). Kaplan-Meier curves showed that the 28-day cumulative survival rate was significantly lower in the Q4 group compared to the Q1 group of S-AKI patients (log-rank test, χ^2^ = 381.5, *p* < 0.001). Subsequently, Cox regression with Q1 as the reference revealed that the risk of death gradually increased with ascending BAR quartiles (Q4 vs. Q1: HR = 0.639, 95% CI: 0.579-0.705, *P* < 0.001). Correlation analysis suggested no significant correlation between BAR and other biological indicators. Additionally, subgroup analysis confirmed the stability of the results. The ROC curve demonstrated that BAR had diagnostic advantages over single indicators such as blood urea nitrogen or albumin (*p* < 0.001; *p* < 0.001). A nomogram incorporating multiple factors including BAR was constructed, which outperformed SOFA and SAPS II in predicting in-hospital mortality for S-AKI, demonstrating good discrimination and calibration capabilities.

**Conclusion:**

BAR, as a simple and convenient biomarker, can effectively predict in-hospital mortality in patients with S-AKI, with its elevation positively correlated with an increased risk of death. The rise in BAR is positively associated with an increased 28-day mortality risk in S-AKI patients, and a higher absolute value of BAR indicates a poorer prognosis for S-AKI patients. The nomogram incorporating BAR demonstrates excellent performance in prediction.

## Introduction

The current internationally accepted definition of sepsis is derived from the 2016 Third International Consensus on the Definition of Sepsis and Septic Shock as “life-threatening organ dysfunction resulting from a dysregulated host response to infection” (1). Its high morbidity and mortality pose a severe threat to global patient safety and public health and is a major clinical challenge in acute and critical care medicine (2). With mortality rates exceeding 25%, sepsis is one of the leading causes of death in intensive care unit (ICU) patients, affecting millions of patients worldwide every year (3).

As sepsis progresses, the risk of complications increases dramatically, particularly acute kidney injury (AKI), which occurs in about two-thirds of sepsis patients and puts these patients at high risk of death (4). Sepsis-induced acute kidney injury (S-AKI) is common in ICUs, and its presence can increase the mortality rate of critically ill patients by six to eight times (5). The current treatment strategy for S-AKI focuses on antibiotics combined with symptomatic therapy, and renal replacement therapy is required to control the condition when necessary.

In addition to prolonging hospital stays and increasing financial burden, S-AKI significantly worsens patient prognosis (6, 7). However, early identification and timely intervention in patients with AKI have been shown to have the potential to reverse the course of the disease, thereby reducing AKI-related mortality (8). Thus, early identification of patients at high risk of S-AKI is essential for management strategies in critically ill patients.

Blood Urea Nitrogen (BUN) is the main product of protein metabolism, and its level changes reflect renal function, metabolic status, and nutritional status (9, 10). Albumin (ALB) is not only an indicator of nutritional status. Albumin (ALB) isn’t just an indicator of nutritional status but also has multiple physiological functions such as antioxidant, anti-inflammatory, etc., which are essential for maintaining homeostasis (10, 11) In inflammation, increased microvascular permeability leads to an imbalance between intravascular and extravascular albumin distribution, decreasing serum albumin concentration in critically ill patients (12).

In recent years, the blood urea nitrogen to serum albumin ratio (BAR), an emerging prognostic marker for inflammation, has become an effective tool for predicting the prognosis of critically ill patients. This is due to its combination of nutritional and renal status and its ease of accessibility. The BAR has been the subject of extensive study and demonstrated to correlate significantly with the prognosis of a variety of diseases, including pneumonia (13), chronic obstructive pulmonary disease (COPD) (14), coronavirus disease-19 (15), gastrointestinal bleeding (16), and heart failure (17). In particular, BAR has also been proven to be an effective predictor of mortality in patients with sepsis (18). Moreover, it has been identified as a significant predictor of increased all-cause mortality in AKI patients, indicating its potential utility in identifying AKI patients at high risk of mortality (19).

Nevertheless, there is a dearth of empirical evidence regarding BAR’s predictive capacity regarding the prognosis of S-AKI patients. In light of the limitations above, the present study sought to elucidate the relationship between baseline BAR levels and the prognosis of patients with S-AKI. This was carried out to provide clinicians with a reliable basis for the early identification of high-risk patients, with the ultimate goal of improving patient outcomes.

## Materials and methods

### Population and data source

The present study is a retrospective analysis of a cohort of patients who met the pre-specified inclusion criteria in the Medical Information Mart for Intensive Care (MIMIC-IV, v2.0) database, an essential resource in critical care. The MIMIC-IV database was constructed and used with the formal approval of the Institutional Review Boards of Beth Israel Deaconess Medical Center and the Massachusetts Institute of Technology. The construction and utilization of the MIMIC-IV database, an invaluable resource in the field of critical care, were formally approved by the institutional review boards of Beth Israel Deaconess Medical Center and the Massachusetts Institute of Technology. A research team member was granted legal access to the database with the requisite authorization (ID number 59839198). As the data were obtained from a publicly accessible source, all protected health information in the MIMIC database was de-identified to safeguard patient privacy (20, 21), negating the necessity for informed consent or ethical approval.

Inclusion criteria for the study population were as follows: 1. Diagnosis of sepsis was following sepsis-3.0 with specific criteria of Sequential organ failure assessment (SOFA) score ≥ 2 and infection or suspected infection. 2. Diagnosis and staging of acute kidney injury (AKI) was based on the 2012 KDIGO Guidelines: an increase in serum creatinine (Serum Creatinine) level of 0.3 mg/dL within 48 h or an increase in SCR level to 1.5 times the baseline SCR level within the past 7 days.

The exclusion criteria were as follows: 1. Age < 18 years; 2. Duration of ICU stay < 24 h; 3. Presence of chronic renal insufficiency reaching CDK stage 5; 4. Patients who had received albumin infusion within 3 days prior to ICU admission; 5. Lack of data on blood urea nitrogen and albumin values.

We collected the following clinical data including gender, age, vital signs (heart rate, systolic blood pressure, diastolic blood pressure, mean arterial pressure), SOFA score, SAPS II (Simplified Acute Physiology Score II).

Comorbidities included Coronary Heart Disease, Diabetes, Chronic Obstructive Pulmonary Disease, hypertension, Cerebrovascular Disease, Chronic Renal Insufficiency, and Chronic Renal Insufficiency Disease.

Interventions included transfuse blood (red blood cells, plasma, and platelets), vasoactive drugs, mechanical ventilation (MV), Renal Replacement Therapy (RRT) from day 1, Continuous Renal Replacement Therapy (CRRT) during ICU stay and other interventions.

Laboratory parameters included White Blood Cell (WBC), Red Blood Cell (RBC), Mean Red Blood Cell Volume (MCV), Hemoglobin (HB), Hematocrit, Platelet (PLT), Lymphocyte, RBC Distribution Width (RDW), Anion Gap (ANG). Platelet (PLT), Lymphocyte (Lymphocyte), RBC Distribution Width (RDW), Anion Gap (Anion Gap), Creatinine (Cr), Prothrombin Time (PT), Glucose (Glucose), Lymphocyte (Lymphocyte), Hemoglobin (HB), Hematocrit (Hematocrit), Platelet, Lymphocyte, RBC Distribution Width (RDW), Anion Gap (Anion Gap), Prothrombin Time (PT), Glucose (glucose, calcium, sodium, potassium, lactate, bilirubin, blood urea nitrogen, albumin, ALB; all laboratory results were recorded in the first evaluation of the patient after admission. All laboratory results were recorded in the patient’s first assessment after admission, from the first 6 h before ICU admission to the first 24 h after ICU admission.

### Groups and endpoints

In this study, the primary endpoint was the 28-day prognosis of sepsis-associated acute kidney injury (S-AKI) patients admitted to the ICU who met the inclusion criteria. Based on their 28-day prognosis, all patients were divided into a survival group and a non-survival group. Additionally, patients were categorized into four groups (Q1, Q2, Q3, Q4) based on the quartile distribution of the absolute value of the blood urea nitrogen-to-albumin ratio (BAR).

The BAR was calculated as the absolute value of blood urea nitrogen divided by the absolute value of albumin.

### Statistical analysis

#### Preliminary analysis and variable selection

##### Data pre-processing

Exclude indicators with more than 40% missing data from the original dataset. For data with less than 40% missing values, fill in any gaps using the random forest algorithm implemented in the mice (22) function (*m* = 5, maxit = 5) of the mice R package. Baseline Characteristic Comparison: In the description of overall characteristics, we use the Kolmogorov-Smirnov test to assess whether the data follow a normal distribution. Continuous data that follow a normal distribution are presented as mean ± standard deviation (μ ± σ) and compared between groups using Student’s *t*-test. Continuous data that do not follow a normal distribution are presented as median (interquartile range) [M (QL, QU)] and compared using the Mann-Whitney *U*-test. Categorical data are presented as proportions (%) and compared using the χ^2^ test.

##### Association analysis of BAR with 28-day mortality risk

Using Cox Proportional Hazards Model: Analyze the relationship between BAR and 28-day mortality risk, reporting the hazard ratio (HR) and 95% confidence interval (95% CI). The proportional hazards assumption of the Cox regression model was simultaneously evaluated using Schoenfeld residuals. A non-significant *p*-value (> 0.05) in the global test would indicate that the proportional hazards assumption was met.

##### BAR grouping and survival analysis by quartiles

Divide BAR into four quartiles (Q1, Q2, Q3, Q4) and use GraphPad Prism version 9.4.1 to perform Kaplan-Meier survival analysis to compare the 28-day cumulative survival rates among the four groups. Simultaneously, use the log-rank test to statistically analyze the differences in survival rates among the four groups. Subsequently, perform Cox regression with Q1 (lowest quartile) as the reference for comparison.

##### Non-linear relationship verification

Use restricted cubic splines (RCS) to analyze the non-linear association between BAR and mortality risk, with the median BAR of all patients as the reference point, and define non-linear effects (P-non-linear < 0.05 as significant).

##### ROC curve plotting

With 28-day prognosis as the dependent variable, plot ROC curves for BAR, BUN, and ALB, and use the DeLong test to formally compare the differences in their AUCs. Simultaneously, plot ROC curves for BAR’s prediction of ICU mortality and in-hospital mortality, and compare them with 28-day mortality.

##### Variable correlation and collinearity handling correlation analysis

Assess the correlation between BAR and other biomarkers using Spearman correlation coefficients and plot a heatmap to display the correlation matrix.

##### Subgroup analysis

Stratify by key treatment measures such as mechanical ventilation and CRRT to assess the heterogeneity of BAR’s association with mortality risk.

#### Prediction model construction and validation

##### LASSO regression variable selection

Based on least absolute shrinkage and selection operator (LASSO) regression analysis, select variables related to 28-day mortality. Choose the lambda (λ) value at the first standard error (se) where λ0.1se is calculated.

##### Logistic regression

Include variables selected by LASSO in univariate and multivariate logistic regression, with results presented as odds ratios (OR) and 95% confidence intervals (CI).

##### Nomogram validation

Construct nomogram models with and without BAR, and compare AUC differences to assess the incremental predictive value of BAR. Calibration and Decision Curve: Evaluate the model’s calibration and clinical utility through calibration curves and decision curve analysis (DCA).

Data analysis was performed using SPSS software version 26.0 and R language version 4.3.2. A two-tailed *P*-value < 0.05 was considered statistically significant.

## Results

### Participants and characteristic

**FIGURE 1:**
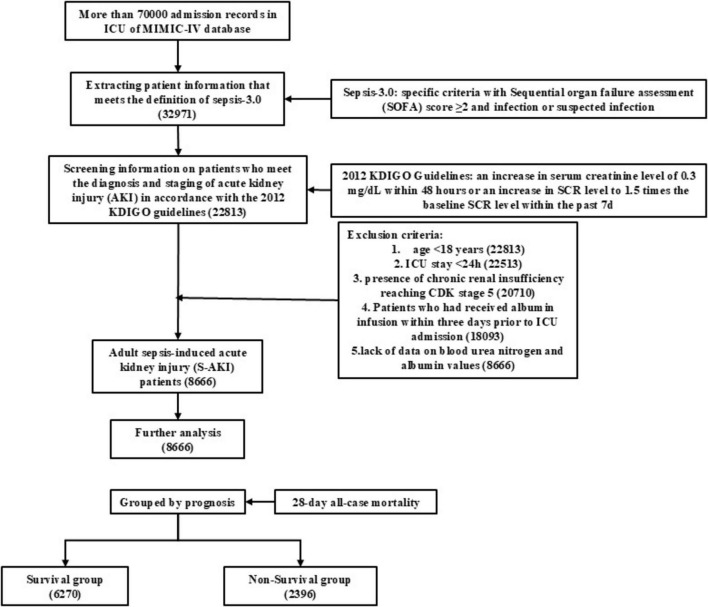
The flowchart of patients screening.

Ultimately, 8,666 eligible patients were enrolled, as illustrated in Figure 1. A comparison of the clinical data of the two groups of patients was presented in Table 1. The mean age was 67.33 years, the mean SOFA score is three, and the mean SAPS II score is 42. The variables of age, heart rate, SOFA score, SAPS II score, WBC, MCV, RDW, anion gap, creatinine, PT, blood potassium, lactate, bilirubin, blood urea nitrogen, and BAR exhibited higher values in the death group (*P* < 0.05). Additionally, the death group showed a higher proportion of patients undergoing treatment or investigations (vasoactive drugs, mechanical ventilation, renal replacement therapy, continuous renal replacement therapy) and complications (coronary heart disease, diabetes mellitus, cerebrovascular disease, chronic renal insufficiency) (*p* < 0.05), as illustrated in Table 1.

**TABLE 1:** Comparison of baseline characteristics between two groups segmented by 28-day mortality.

Variables		All patients (*n* = 8,666)	Survival group (*n* = 6,270)	Non-survival group (*n* = 2,396)	t/Z/χ^2^	*P*-value
Genders	F	3,666 (42.3%S)	2,632 (42.0 per cent)	1,034 (43.2 per cent)		
	M	5,000 (57.7 per cent)	3,638 (58.0 per cent)	1,362 (56.8%)	χ^2^ = 0.985	0.331
Age		67.33 (56.26-78.93)	65.94 (54.80-77.34)	71.52 (59.97-82.63)	Z = −13.561	<0.001
Heart rate		88.83 (76.96-101.37)	87.86 (76.30-100.13)	91.49 (78.95-104.00)	Z = −7.569	<0.001
SBP		111.28 (103.04-122.70)	112.28 (104.00-123.85)	108.39 (100.83-119.96)	Z = −10.565	<0.001
DBP		60.65 (54.65-67.71)	61.08 (55.08-68.04)	59.34 (52.97-66.58)	Z = −7.293	<0.001
MAP		74.67 (68.90-81.81)	75.28 (69.68-82.36)	73.10 (67.24-80.18)	Z = −9.788	<0.001
Coronary heart disease	Have	2860 (33.0 per cent)	2015 (32.1%)	845 (35.3 per cent)		
	None	5806 (67.0 per cent)	4255 (67.9 per cent)	1551 (64.7 per cent)	χ^2^ = 7.681	0.006
Diabetes	Have	2824 (32.6 per cent)	2100 (33.5 per cent)	724 (30.2 per cent)		
	None	5842 (67.4)	4170 (66.5 per cent)	1672 (69.8%)	χ^2^ = 8.468	0.004
COPD	Have	816 (9.4 per cent)	573 (9.1%)	243 (10.1%)		
	None	7850 (90.6 per cent)	5697 (90.9 per cent)	2153 (89.9 per cent)	χ^2^ = 2.045	0.162
Cerebrovascular disease	Have	584 (6.7 per cent)	384 (6.1%)	200 (8.3 per cent)		
	None	8082 (93.3)	5886 (93.9 per cent)	2196 (91.7 per cent)	χ^2^ = 13.629	<0.001
Hypertensive	Have	5438 (62.8%)	3953 (63.0 per cent)	1485 (62.0 per cent)		
	None	3228 (37.2 per cent)	2317 (37.0 per cent)	911 (38.0 per cent)	χ^2^ = 0.846	0.358
Chronic renal insufficiency	Have	2089 (24.1)	1453 (23.2 per cent)	636 (26.5 per cent)		
	None	6577 (75.9 per cent)	4817 (76.8%)	1760 (73.5 per cent)	χ^2^ = 10.764	<0.001
Transfuse blood	Have	268 (3.1%)	185 (3.0 per cent)	83 (3.5 per cent)		
	None	8398 (96.9 per cent)	6085 (97.0 per cent)	2313 (96.5 per cent)	χ^2^ = 1.526	0.213
Vasoactive drug	Have	4050 (46.7 per cent)	2741 (43.7 per cent)	1309 (54.6 per cent)		
	None	4616 (53.3 per cent)	3529 (56.3 per cent)	1087 (45.4 per cent)	χ^2^ = 82.99	<0.001
Mechanical ventilation	Have	5436 (62.7 per cent)	3802 (60.6 per cent)	1634 (68.2 per cent)		
	None	3230 (37.3 per cent)	2468 (39.4 per cent)	762 (31.8%)	χ^2^ = 42.367	<0.001
RRT	Have	469 (5.4 per cent)	276 (4.4 per cent)	193 (8.1%)		
	None	8197 (94.6 per cent)	5994 (95.6 per cent)	2203 (91.9 per cent)	χ^2^ = 45.195	<0.001
CRRT	Have	760 (8.8%)	387 (6.2 per cent)	373 (15.6 per cent)		
	None	7906 (91.2 per cent)	5883 (93.8%)	2023 (84.4 per cent)	χ^2^ = 191.263	<0.001
WBC		11.70 (7.80-16.90)	11.40 (7.70 -16.50)	12.40 (8.20-18.30)	Z = −5.747	<0.001
RBC		3.60 (3.00-4.22)	3.65 (3.06-4.26)	3.44 (2.86-4.10)	Z = −8.403	<0.001
MCV		92.00 (88.00-97.00)	92.00 (88.00-97.00)	94.00 (89.00-99.00)	Z = −8.306	<0.001
HB		10.70 (9.00-12.50)	10.90 (9.10-12.70)	10.30 (8.60-12.20)	Z = −7.969	<0.001
Hematocrit		33.00 (27.90-38.30)	33.40 (28.30-38.60)	32.10 (27.10-37.68)	Z = −6.017	<0.001
PLT		195.00 (125.00-277.00)	199.00 (131.00-279.00)	183.00 (109.00-269.00)	Z = −5.945	<0.001
Lymphocytes		0.94 (0.55-1.51)	0.97 (0.58-1.53)	0.88 (0.49-1.45)	Z = −5.026	<0.001
RDW		15.30 (14.00-17.20)	15.10 (13.80-16.90)	16.00 (14.40-18.20)	Z = −14.789	<0.001
Anion gap		16.00 (13.00-20.00)	16.00 (13.00-19.00)	17.00 (14.00-21.00)	Z = −9.991	<0.001
CR		1.20 (0.80-1.90)	1.20 (0.80-1.80)	1.40 (0.90-2.20)	Z = −10.201	<0.001
PT		14.90 (12.80-19.50)	14.50 (12.60-18.30)	16.30 (13.50-22.70)	Z = −15.090	<0.001
Glucose		134.00 (106.00-180.00)	135.00 (107.00-180.00)	131.00 (102.00-180.00)	Z = −3.402	0.001
Calcium		8.40 (7.80-9.00)	8.40 (7.80-9.00)	8.30 (7.70-8.98)	Z = −2.713	0.007
Sodium		138.00 (134.00-141.00)	138.00 (135.00-141.00)	138.00 (133.00-141.00)	Z = −2.742	0.006
Potassium		4.30 (3.80-4.90)	4.20 (3.80-4.80)	4.40 (3.80-5.08)	Z = −6.449	<0.001
Lactate		2.10 (1.40-3.40)	2.00 (1.40-3.10)	2.50 (1.70-4.20)	Z = −15.064	<0.001
Bilirubin		0.70 (0.40-1.80)	0.70 (0.40-1.70)	0.80 (0.40-2.50)	Z = −7.057	<0.001
BNU		27.00 (17.00-44.00)	24.00 (16.00-39.25)	33.00 (21.00-53.00)	Z = −9.788	<0.001
ALB		3.10 (2.60-3.50)	3.10 (2.70-3.60)	2.90 (2.40-3.40)	Z = −13.766	<0.001
BAR		8.93 (5.29-15.22)	7.94 (4.86-13.55)	11.98 (7.14-19.66)	Z = −20.075	<0.001
SOFA		3.0 (2.0-5.0)	3.0 (2.0-5.0)	4.0 (2.0-6.0)	Z = −11.497	<0.001
SAPS II		42.0 (33.0-52.0)	40.0 (32.0-49.0)	50.0 (41.0-60.0)	Z = −28.748	<0.001

SBP, Systolic Blood Pressure; DBP, Diastolic Blood Pressure; MAP, Mean Arterial Pressure; SOFA, Sequential Organ Failure Assessment; SAPS II, Simplified Acute Physiology Score II; RRT, Renal Replacement Therapy; CRRT, Continuous Renal Replacement Therapy; WBC, White Blood Cell; RBC, Red Blood Cell; MCV, Mean Red Blood Cell Volume; HB, hemoglobin; PLT, Platelet; RDW, RBC Distribution Width; Cr, Creatinine; PT, Prothrombin Time; BUN, Blood urea Blood urea nitrogen; ALB, Albumin; BAR, Blood urea nitrogen to serum albumin ratio.

### Association analysis of BAR with 28-day mortality risk

A Cox analysis was conducted on BAR, and the results indicated a positive correlation between BAR and the risk of 28-day mortality, with an HR of 1.029 (95% CI 1.026-1.032) (see Table 2). We conducted a formal test of the proportional hazards assumption for the Cox model using Schoenfeld residuals. The overall test resulted in a *p*-value of 0.91 for the continuous BAR variable, indicating no significant deviation from the proportional hazards assumption, confirming that the assumption was not violated and supporting the validity of the Cox model (χ^2^ = 0.0124, *p* = 0.91).

**TABLE 2:** BAR cox regression analysis.

	β	SE	*P*	HR	HR 95% CI	
BAR	0.029	0.001	<0.001	1.029	1.026	1.032

### Kaplan-Meier survival curve

**FIGURE 2:**
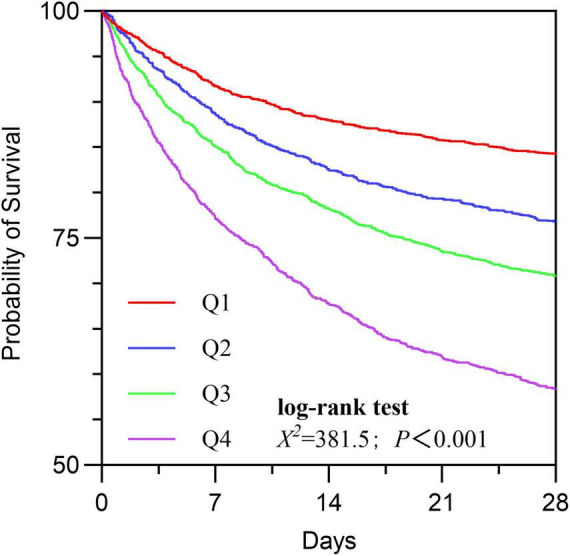
The Kaplan-Meier survival curves were used to compare the 28-day cumulative survival rates of BAR in the Q1, Q2, Q3, and Q4 groups.

Based on the quartiles of BAR, it was divided into four groups: Q1 ≤ 5.29; 5.29 < Q2 ≤ 8.93; 8.39 < Q3 ≤ 15.22; Q4 > 15.22. Subsequently, corresponding Kaplan-Meier survival curves were plotted based on the specific survival times (Figure 2). The results showed that compared with the Q1 group, the 28-day cumulative survival rate of S-AKI patients in the Q4 group was significantly reduced (log-rank test, χ^2^ = 381.5, *p* < 0.001).

Subsequently, Cox regression was performed with Q1 as the reference, and the results indicated that the risk of mortality gradually increased with the elevation of BAR quartiles (Q4 vs. Q1: HR = 0.639, 95% CI: 0.579-0.705, *P* < 0.001) (Table 3).

**TABLE 3:** Cox regression was performed for the Q2, Q3, and Q4 groups with Q1 as the reference.

	β	SE	*P*	HR	HR 95%CI	
Q2	−1.145	0.064	<0.001	0.318	0.281	0.360
Q3	−0.716	0.058	<0.001	0.489	0.436	0.548
Q4	−0.448	0.050	<0.001	0.639	0.579	0.705

### Non-linear association between BAR and all-cause mortality

**FIGURE 3:**
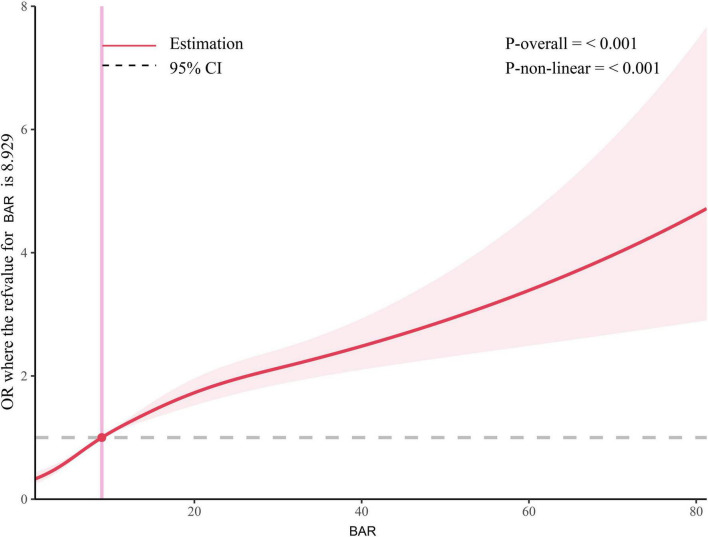
RCS analysis of the relationship between BAR and the risk of 28-day all-cause mortality in S-AKI patients. The shaded area represents the 95% confidence interval.

RCS analysis revealed a non-linear association between BAR and 28-day mortality (P-non-linear < 0.001). Notably, the threshold of BAR ≥ 8.929 (median of the study population) where the risk of mortality significantly increased is data-driven and specific to the study cohort. This threshold should not be interpreted as a clinically validated cut-off value and requires further validation in independent populations (Figure 3).

### ROC curves analyses

**FIGURE 4:**
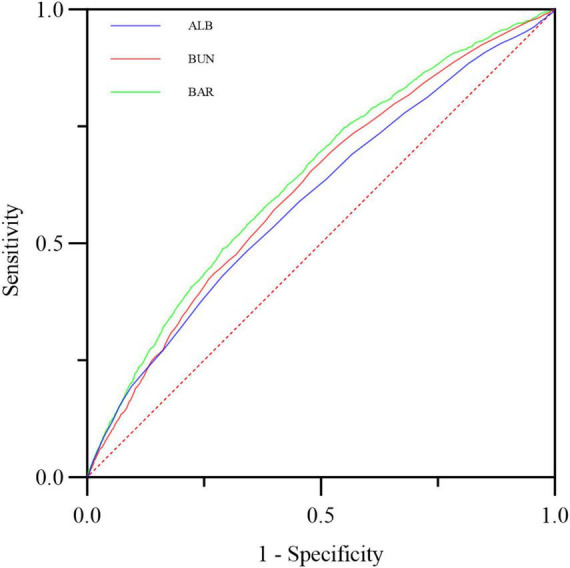
The predictive value of ALB, BUN and BAR for the prognosis of S-AKI in critically ill patients was compared.

The ROC analysis results showed that the optimal cutoff value of BAR for predicting 28-day mortality was 12.36, with an area under the curve (AUC) of 0.639 (95% CI: 0.626-0.652). Furthermore, compared with BUN alone (AUC: 0.617, 95% CI: 0.604-0.630) or ALB alone (AUC: 0.595, 95% CI: 0.582-0.609), BAR demonstrated diagnostic advantage (*P* < 0.001; *P* < 0.001), as shown in Table 4 and Figure 4.

**TABLE 4:** Diagnostic value of BUN, ALB, and BAR in the prediction of all-cause mortality in patients with S-AKI.

Variables	AUC	95%CI	Cutoff	Sensitivity	Specificity	VS BAR Z	VS BAR P
BUN	0.617	0.604–0.630	25.50	0.6536	0.5222	−9.380	<0.001
ALB	0.595	0.582–0.609	2.85	0.4825	0.6600	−5.719	<0.001
BAR	0.639	0.626–0.652	12.36	0.4887	0.7104		

**FIGURE 5:**
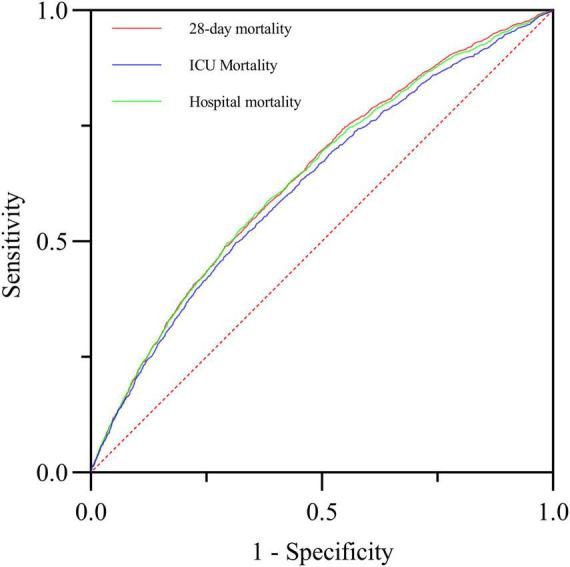
The predictive value of BAR for 28-day mortality, ICU mortality and in-hospital mortality in critically ill patients with S-AKI was compared.

Additionally, we plotted ROC curves to evaluate the predictive ability of BAR for ICU mortality and in-hospital mortality in S-AKI patients. The AUCs were 0.622 (95% CI: 0.606-0.638) and 0.636 (95% CI: 0.623-0.650), respectively. There was no significant difference compared to the AUC of 0.639 (95% CI: 0.626-0.652) for predicting 28-day mortality with BAR, as shown in Figure 5.

### Variable correlation analysis

**FIGURE 6:**
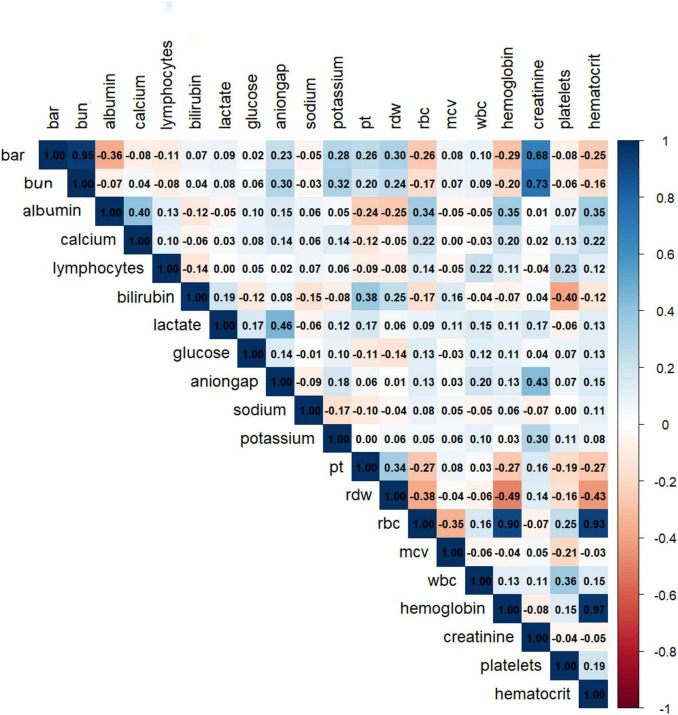
Correlation analysis heatmap.

The correlation analysis results indicated a high correlation between BAR and BUN, with a correlation coefficient *r* = 0.95 > *r* = 0.7. At the same time, the correlation between BAR and other biomarkers was not significant, with correlation coefficients r all less than 0.7 (Figure 6).

### Subgroup analysis

**FIGURE 7:**
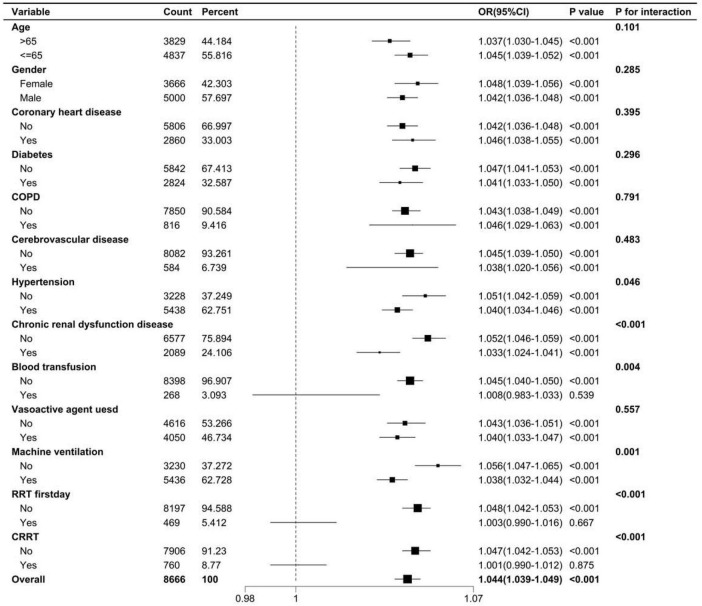
The subgroup analysis between BAR and 28-day all-cause mortality.

To further validate the stability of our results, subgroup analyses were performed in this study to investigate the interaction between BAR and variables that may affect 28-day mortality in patients with S-AKI, as illustrated in Figure 7. The results demonstrated that there was an all-cause mortality of 28-day mortality in patients with S-AKI due to hypertension, chronic renal insufficiency (CKD5 stage not reached), blood transfusion, mechanical ventilation, RRT, CRRT, and BAR interaction (interaction *p* < 0.05). No significant interaction was observed in the other subgroups (*p* > 0.05).

### Least absolute shrinkage and selection operator regression analysis

**FIGURE 8:**
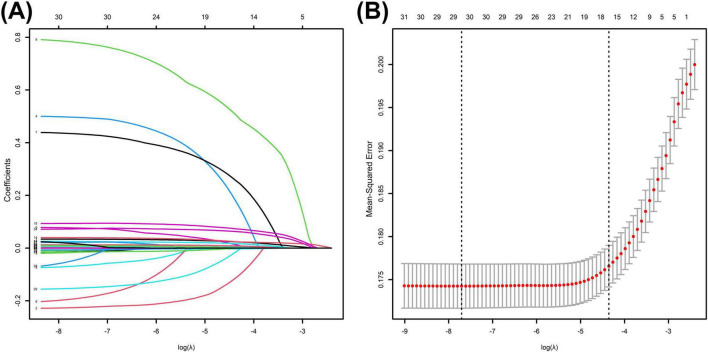
**(A)** Selection process of prognostic variables of S-AKI by LASSO regression. **(B)** Selection process of the value of lambda by cross validation.

Using 28-day prognosis as the dependent variable, we conducted a 10-fold cross-validation LASSO regression analysis with the 29 variables from Table 1 that had *p* < 0.05, excluding the scoring systems and BUN and ALB. The lambda.1se was calculated to be 0.01. Ultimately, 16 variables were selected, including BAR, age, diabetes, cerebrovascular disease, use of vasoactive drugs, mechanical ventilation, CRRT, heart rate, systolic blood pressure, bilirubin, lactate, PT, RDW, MCV, WCB, and CR, as shown in Figures 8A, B.

### Nomogram incorporated with BAR showed excellent discriminative capacity

**FIGURE 9:**
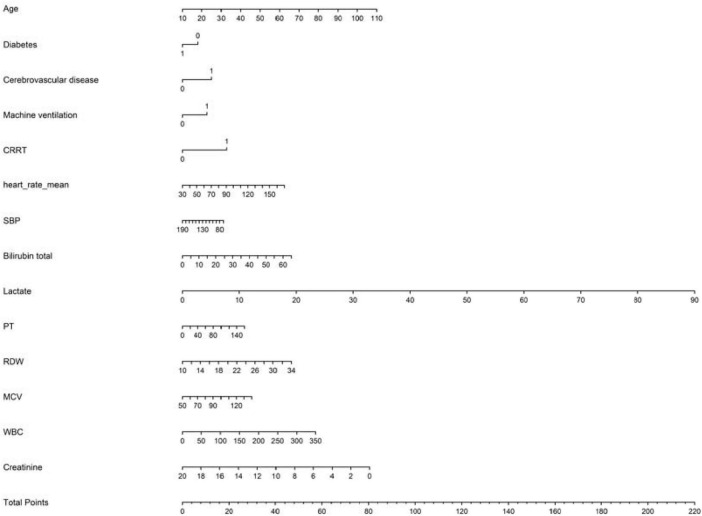
The survival nomogram for predicting in-hospital mortality of S-AKI patients. When using it, drawing a vertical line from each variable upward to the points and then recording the corresponding points. The point of each variable was then summed up to obtain a total score that corresponds to a predicted probability of in-hospital mortality at the bottom of the nomogram

This study ultimately included 15 risk factors, as indicated by the LASSO regression for predicting in-hospital mortality (Figures 8A, B). Additionally, a nomogram for predicting 28-day all-cause mortality in S-AKI patients was constructed based on multivariate logistic analysis (Table 5). Fifteen factors, including BAR, age, heart rate, systolic blood pressure, diabetes, cerebrovascular disease, mechanical ventilation, CRRT, bilirubin, lactate, creatinine, PT, RDW, MCV, and WBC, were ultimately included in the nomogram (Figure 9).

**TABLE 5:** Logistic regression analysis for the risk factors of in-hospital mortality selected by LASSO regression.

Variables	One-factor logistic		Multifactor logistic	
	**OR (95% CI)**	**P**	**OR (95% CI)**	***P***
BAR	1.044 (1.039-1.049)	<0.001	1.040 (1.033-1.047)	<0.001
Age	1.022 (1.019-1.025)	<0.001	1.033 (1.029-1.037)	<0.001
Heart rate	1.011 (1.008-1.013)	<0.001	1.012 (1.009-1.015)	<0.001
SBP	0.985 (0.981-0.988)	<0.001	0.995 (0.991-0.999)	0.011
Diabetes	1.163 (1.050-1.288)	0.004	1.306(1.167-1.461)	<0.001
Cerebrovascular disease	0.716 (0.600-0.856)	<0.001	0.609 (0.501-0.741)	<0.001
Vasoactive drug	0.645 (0.587-0.709)	<0.001	0.936 (0.833-1.052)	0.266
Machine ventilation	0.718 (0.650-0.794)	<0.001	0.670 (0.597-0.753)	<0.001
CRRT	0.357 (0.307-0.415)	<0.001	0.477 (0.398-0.571)	<0.001
Bilirubin	1.042 (1.033-1.050)	<0.001	1.029 (1.019-1.039)	<0.001
Lactate	1.132 (1.111-1.152)	<0.001	1.099 (1.078-1.122)	<0.001
Creatinine	1.109 (1.075-1.145)	<0.001	0.852 (0.809-0.898)	<0.001
Pt	1.017 (1.013-1.020)	<0.001	1.007 (1.003-1.010)	<0.001
RDW	1.128 (1.109-1.147)	<0.001	1.080 (1.059-1.102)	<0.001
MCV	1.025 (1.019-1.031)	<0.001	1.013 (1.007-1.020)	<0.001
WBC	1.013 (1.008-1.018)	<0.001	1.006 (1.002-1.011)	0.008

BAR, continuous renal replacement therapy; CRRT, systolic blood pressure; SBP, P T, prothrombin time; RDW, RBC distribution width; MCV, mean red blood cell volume; WBC, white blood cell.

**FIGURE 10:**
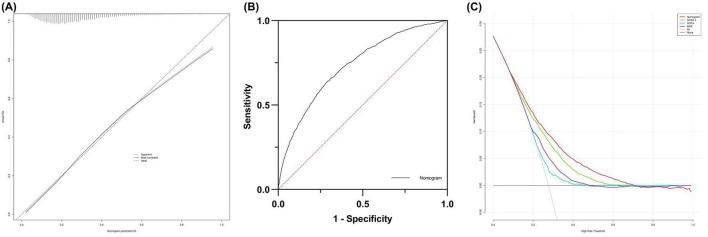
**(A)** The calibration curve for predicting in-hospital mortality. **(B)** Receiver operating characteristic curve analysis of blood urea nitrogen to serum albumin ratio and nomogram for in-hospital mortality prediction. **(C)** Decision curve analysis DCA of the nomogram to predict in-hospital mortality.

The calibration curve of the prediction nomogram demonstrated significant consistency between the actual and predicted probabilities (Figure 10A). Furthermore, the AUC of the prediction nomogram was 0.734 (95% CI: 0.722–0.745), indicating good predictive ability for in-hospital mortality (Figure 10B). Ultimately, the clinical utility of the prediction nomogram was determined through the application of decision curve analysis (DCA), which demonstrated that the nomogram was helpful in decision-making (Figure 10C).

**FIGURE 11:**
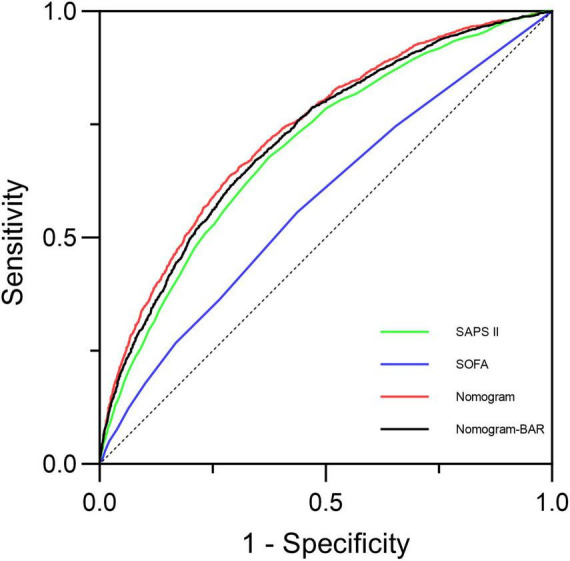
The discrimination performance of the newly developed prediction model was compared with the nomogram model with BAR removed and SOFA and SAPS II.

For a long time, the SOFA and SAPS II scores have been classic tools for assessing the severity of sepsis. We compared our newly developed nomogram (AUC = 0.734, 95% CI: 0.722–0.745) with the SOFA (AUC = 0.578, 95% CI: 0.564-0.591) and SAPS II (AUC = 0.699, 95% CI: 0.687-0.711) scores to evaluate its performance and potential applications. Additionally, to explore the weight of BAR in the nomogram, we plotted the ROC curve of the nomogram without BAR and compared its AUC with that of the original nomogram. The results showed that the discriminative ability of the nomogram was significantly higher than that of the other two scores and the nomogram without BAR, as illustrated in Figure 11 and Table 6.

**TABLE 6:** Delong’s test for comparison different models.

Comparison	AUC	*Z*	*P*
Nomogram vs. SOFA	0.734 vs. 0.578	19.765	<0.001
Nomogram vs. SAPS II	0.734 vs. 0.699	5.652	<0.001
SOFA vs. SAPSII	0.578 vs. 0.699	−15.655	<0.001
Nomogram vs. BAR	0.734 vs. 0.639	6.418	<0.001
Nomogram vs. BUN	0.734 vs. 0.617	17.268	<0.001
Nomogram vs. ALB	0.734 vs. 0.595	32.134	<0.001
Nomogram vs. nomogram-BAR	0.734 vs. 0.720	6.418	<0.001

## Discussion

To our knowledge, this study is the first to explore the predictive role of BAR in in-hospital mortality among patients with sepsis-associated acute kidney injury (S-AKI). BAR is an independent risk factor for 28-day mortality in S-AKI patients. The Kaplan-Meier survival curve concluded that the 28-day cumulative survival rate was significantly lower in the Q4 group compared to the Q1 group of S-AKI patients (log-rank test, χ^2^ = 381.5, *p* < 0.001). Cox regression analysis, with Q1 as the reference, indicated that the risk of death gradually increased with increasing BAR quartiles (Q4 vs. Q1: HR = 0.639, 95% CI: 0.579-0.705, *p* < 0.001). Importantly, the proportional hazards assumption of the Cox model was rigorously tested and validated through Schoenfeld residuals, ensuring the reliability of our hazard ratio estimates. RCS analysis revealed a non-linear relationship between BAR and the risk of 28-day all-cause mortality in S-AKI patients. It is important to emphasize that the threshold of BAR ≥ 8.929 identified in this study, while statistically significant, is dependent on the characteristics of the study population and should not be directly applied to clinical practice without further validation. Furthermore, the ROC curve demonstrated that BAR had a diagnostic advantage over using blood urea nitrogen (BUN) or albumin alone for predicting the prognosis of S-AKI patients (*p* < 0.001). Correlation analysis showed a high correlation between BAR and BUN, with a correlation coefficient *r* = 0.95 > 0.7. At the same time, the correlation between BAR and other biomarkers was not significant, with correlation coefficients r all less than 0.7. Subgroup analysis further confirmed the stability of the study results. Additionally, the nomogram combining BAR with other prognostic factors demonstrated significantly higher ability to predict in-hospital mortality compared to SOFA and SAPS II, exhibiting excellent discrimination and calibration capabilities.

The etiological and pathophysiological mechanisms underlying the relationship between BAR and poor prognosis remain unknown. By considering the four conditions of malnutrition, dehydration, liver reserve, and renal reserve, BAR can be assessed as a composite body reserve and may prove to be a more useful indicator of disease severity than BUN or ALB. Also recently, BAR has been employed as a novel biomarker to evaluate the prognosis of diverse diseases and is a crucial prognostic indicator for mortality in numerous conditions, including lung cancer, gastrointestinal hemorrhage, and community-acquired pneumonia (16, 23, 24). The present study reports a robust correlation between BAR levels and 28-day mortality in S-AKI patients, with a high predictive value.

This study presents an innovative column-line graphical model that incorporates BAR, a core predictor, which demonstrates high discrimination and good calibration for the prediction of mortality in S-AKI patients. In addition to BAR, the model incorporated several other factors, including age, heart rate, systolic blood pressure, history of diabetes mellitus, cerebrovascular disease, need for mechanical ventilation, use of continuous renal replacement therapy (CRRT), bilirubin level, lactate level, and creatinine concentration. The columnar graphical model incorporates several key variables, including plasminogen time (PT), red blood cell distribution width (RDW), mean corpuscular volume (MCV), and white blood cell count (WBC). These have been demonstrated to significantly correlate with in-hospital mortality in patients with S-AKI, and thus represent a crucial component of the model.

The results of this study demonstrate that the age of patients in the death group is significantly higher than that of the survival group, and this difference is statistically significant. This finding aligns with previous reports in the literature indicating that the elderly population is more susceptible to acute kidney injury (AKI) and has a poor prognosis (25). AKI is a disease that occurs more frequently and has a poor prognosis in the elderly population. As sepsis progresses to septic shock, patients often develop hemodynamic instability, as evidenced by increased heart rate and decreased systolic blood pressure. This can act synergistically on the heart and kidneys, leading to cardiac pumping insufficiency and renal insufficiency, which exacerbate systemic inflammation and multiorgan dysfunction.

Regarding the relationship between type 2 diabetes and sepsis, numerous studies have indicated that although it does not directly elevate the overall mortality rate in septic patients, it markedly increases the susceptibility to S-AKI (26, 27). Additionally, chronically elevated blood glucose levels have been observed to directly damage renal tissue by activating pivotal signaling pathways, such as NF-κB and TGF-β, and inducing oxidative stress. An observational study conducted by Basi and colleagues further substantiated the notion that insulin resistance with an elevated risk of hyperglycemia is significantly associated with an increase in the in-hospital mortality rate of patients with acute renal failure (28). The mechanism behind the phenomenon of hypoglycemia, which may serve as a marker of disease severity, involves a dramatic increase in the demand for glucose in macrophage-enriched tissues (e.g., intestines, liver), a process that may exacerbate the condition and elevate the risk of death (29). Acute ischemic stroke, a common cerebrovascular disease, not only damages the nervous system but is often associated with AKI, increasing the risk of poor prognosis. It has been demonstrated that approximately 11.60% of patients who have experienced an ischemic stroke also suffer from acute kidney injury (AKI), which markedly elevates the likelihood of unfavorable short- and long-term outcomes (30).

Our study has identified mechanical ventilation with continuous renal replacement therapy (CRRT) as an independent risk factor for in-hospital mortality in patients with sepsis-associated acute kidney injury (S-AKI). A review of the literature revealed that invasive mechanical ventilation was a significant risk factor for AKI (31) and that ICU mortality was higher in septic mechanically ventilated patients than in non-septic patients (32). Prolonged mechanical ventilation or the use of improper ventilation strategies may result in the development of serious complications, including lung injury, airway infection, and pneumatic pressure injury. Clinical data indicate that abnormal parameters, including hypoxemia, hypercapnia, elevated positive end-expiratory pressure (PEEP) levels, and high tidal volumes, are significant predictors of acute kidney injury (AKI) in mechanically ventilated patients (31). Previous studies have also corroborated the robust correlation between mechanical ventilation and the incidence of AKI, risk factors, all-cause mortality, and renal prognosis (33). CRRT is becoming an increasingly prevalent strategy for the management of AKI in critically ill patients (34). Early initiation of continuous renal replacement therapy (CRRT) has been demonstrated to not only improve renal function and reduce inflammation but also to significantly reduce mortality. Zhang et al. have shown that CRRT can lead to a significant reduction in parathyroid hormone and renin levels, optimize calcium and phosphorus metabolism, and thus improve the quality of life of patients (35). Nevertheless, it is important to acknowledge that recent studies have indicated that CRRT may potentially contribute to increased all-cause mortality in specific instances (36).

It is widely accepted that the antioxidant and anti-inflammatory properties possessed by bilirubin at low total bilirubin levels may be associated with an increased susceptibility to specific diseases. It has been definitively demonstrated that an elevation in serum bilirubin levels during the initial 4 days following hospital admission is markedly correlated with an increased mortality rate in patients with severe sepsis (37). Moreover, the extant evidence points toward a potential biological link between bilirubin and acute kidney injury (AKI) (38). A retrospective cohort study conducted by Chunlei Gong et al. included 655 patients with sepsis and provided a comprehensive analysis of the relationship between serum lactate levels and S-AKI morbidity and mortality. The study revealed that elevated serum lactate levels were independent predictors of S-AKI morbidity and mortality. Specifically, the risk of AKI in patients with sepsis increased to 1.772-fold when the serum lactate concentration reached or exceeded 2.75 mmol/L. Furthermore, when the serum lactate concentration increased to 5.95 mmol/L or higher, the risk of in-hospital mortality in patients with S-AKI increased to 1.511-fold (39). Creatinine, a small molecule produced by muscle metabolism, is widely used as a functional biomarker for AKI. Typical features of AKI include a dramatic increase in serum creatinine levels, a decrease in urine output, or both, which highlights the value of creatinine in the early diagnosis of AKI (40). Creatinine has long been a key diagnostic, monitoring, and prognostic indicator in renal disease. There is a statistically significant correlation between creatinine levels and disease progression and mortality in SAKI (41).

The measurement of prothrombin time (PT), a pivotal indicator for evaluating the capacity of blood to coagulate, reflects the duration of blood coagulation and is indispensable for elucidating the body’s capability to form blood clots (42). The pathological evolution of sepsis is characterized by dynamic changes in the levels of coagulation factors, platelets, and endothelial cells, which can significantly interfere with the coagulation cascade. This leads to prolongation of PT, which is strongly associated with poor prognosis (43). The data from the present study further corroborate the hypothesis that PT is significantly prolonged in patients with S-AKI (sepsis-associated acute kidney injury) with a poor prognosis. This suggests that PT prolongation may be a potential prognostic factor for a poor prognosis in S-AKI.

Recent studies have demonstrated that Red Cell Distribution Width (RDW) has become an independent and significant prognostic marker in critically ill patients with Acute Kidney Injury (AKI) and that elevated levels of RDW frequently indicate an increased risk of mortality in these patients (44). It is frequently observed that elevated levels of RDW are indicative of an elevated risk of mortality in patients presenting with these conditions. Furthermore, the incidence of sepsis is also significantly associated with RDW levels. These levels not only reflect the degree of underlying inflammatory response in sepsis patients, but they also show promise as a prognostic marker for mortality, with satisfactory discriminatory efficacy in predicting in-hospital mortality, which is a valuable reference for the prognostic assessment of sepsis patients (45). It is noteworthy that this study also reveals an association between elevated mean corpuscular volume (MCV) and increased risk of death in patients with S-AKI, indicating that MCV may serve as an independent predictor of 28-day mortality risk in patients with S-AKI. Additionally, elevated white blood cell count (WBC) was identified as a significant predictor of mortality (*p* < 0.001), aligning with recent literature on the subject (46), this further underscores the pivotal role of these laboratory markers in evaluating the prognosis of S-AKI patients.

This study has several notable strengths. Firstly, the correlation between BAR and short-term prognosis is explored, and subgroup analyses are also performed to investigate the interaction between BAR and variables that may affect the short-term prognosis of patients with S-AKI. This demonstrates the stability of the results. The study also benefited from a large sample size of 8,666 cases, which enhances the reliability of the results. Also, in addition to the traditional multivariate logistic regression, the LASSO regression is employed to enhance the accuracy and reliability of variable selection. However, there are also some limitations that need to be considered. This study should address several constraints. Firstly, this study is a retrospective analysis based on a single public database, inherently introducing potential bias. Therefore, external validation in an independent cohort is necessary to confirm the generalizability of our findings. Secondly, although our study employed LASSO regression to mitigate overfitting, the data-driven nature of the predictive model may still pose a risk of over-optimization. Additionally, during the data cleaning process, missing data were imputed using the Random Forest algorithm to minimize bias; however, the imputed values are estimates and may introduce a certain degree of uncertainty into the analysis, especially for variables with complex interactions. The potential impact of missing data and the imputation method on the results should be carefully considered. Thus, future studies require prospective designs and complete data collection. Thirdly, when evaluating the prognostic value of the blood urea nitrogen to albumin ratio in patients with S-AKI, it is essential to consider the overall condition of the patient, as the levels of both urea nitrogen and albumin can be influenced by a multitude of factors. For patients with liver cirrhosis, albumin levels may generally be low due to hepatic dysfunction. Although this study did not specifically analyze this patient population in detail, the BAR ratio, as a prognostic marker that combines renal function and nutritional status, may still theoretically have certain reference value. However, for patients with conditions such as liver cirrhosis that can lead to severe hypoalbuminemia, the interpretation of the BAR ratio may require more caution and should be comprehensively evaluated in conjunction with the patient’s specific condition and other relevant indicators. Future studies can further explore the applicability and predictive value of the BAR ratio in this patient population. Fourthly, it is noteworthy that the current study only analyzed the relationship between the BAR value at ICU admission and patient prognosis, without exploring the dynamic changes of the BAR value during the course of the disease and its potential impact on patient outcomes. As a composite indicator of renal function and nutritional status, the BAR value may more sensitively reflect changes in a patient’s condition over time. Therefore, in future studies, we will attempt to collect multiple BAR values from patients to analyze the trend of its changes during the disease course and explore the relationship between these changes and patient prognosis. This will help us to gain a more comprehensive understanding of the role of the BAR value in prognostic assessment of patients with sepsis-associated acute kidney injury and provide more precise evidence for clinical decision-making.

## Conclusion

BAR, as a readily available biomarker, can predict in-hospital mortality in patients with sepsis-associated acute kidney injury (S-AKI). The evidence provided by this study demonstrates that BAR is effective in predicting in-hospital mortality in S-AKI patients, with an increase in BAR positively correlated with an increased risk of death. An elevation in BAR is positively associated with an increased 28-day mortality risk in S-AKI patients, and a higher absolute value of BAR indicates a poorer prognosis for S-AKI patients. A nomogram that combines BAR with other relevant variables exhibits good predictive ability for in-hospital mortality. Clinicians should assess the prognosis of such patients early and increase vigilance accordingly. Furthermore, the significance of BAR for the prognosis of S-AKI patients should be investigated in more prospective trials in the future.

## Data Availability

The datasets presented in this study can be found in online repositories. The names of the repository/repositories and accession number(s) can be found at: https://physionet.org/content/mimiciv/2.2/.
